# Supplementary material for: Machine learning based stellar classification with highly sparse photometry data
Source: Open Res Eur. 2024 Feb 16;4:29. [Version 1] doi: 10.12688/openreseurope.17023.1 (PMC11362725; doi:10.12688/openreseurope.17023.1)
Supplement: Peer review history

# Response

**Published:** 2024-08-14

**Authors:** Seán Enis Cody

**Affiliations:** Know-Center GmbH, Graz, Austria

*This paper has good intentions, however, the work seemed to have started on a bad footing, as the choice and construction of the data set is not very good. The classification of stars utilising XGBoost has some novelty in it, and the sample selection has been detailed. However, here are my issues with the paper:*

- *The paper's objectives are not entirely clear despite obtaining some usable results, and the conclusions are not quantitative enough.*
- *Several parts of the methodology are not very well explained, e.g. the exact inputs were not described, the wrong usage of the concept 'data augmentation', graphs plotted with unclear labels, issues with the preprocessing stage (training data set not enough 'signals' compared to 'background').*
- *Unsatisfactory command of grammar and the incorrect usage of the word 'skill'.*
- *A certain section in the results section could be removed as it is either not explained correctly, used a questionable methodology, or produced results which would not solve or help the case of the paper.*

*All detailed comments on the paper are shown below as part of the comments to the authors. I'm actually prone to rejection of the paper, but then again, the paper's idea has some great novelty, just that it was executed poorly, making it unsatisfying to read. However if the authors are willing to make major changes to the paper detailed below, I think it is worth a second consideration.* **Answer**: Dear Reviewer, thank you for both your time and effort in reading our work and providing us with your feedback. We have changed the presentation of the results (especially the plots) and parts of the discussion. We have also carefully considered your comment on the overall structure of the study. However, we think that this was mainly caused by the fact that the objectives of our research were not made sufficiently clear in the manuscript. We apologize for the confusion, and we have now clarified the objectives. Notwithstanding that other approaches might also be valuable; we are convinced that the current approach of the study is the best way to reach those objectives. You will find a detailed discussion on this in our point-by-point responses below. Finally, we have given the paper another round of careful proofreading, with special focus on grammar. *The following are my comments on the paper, according to the different sections:* *Abstract:*  

- *The "which variables should be used" statement should be replaced with a sentence indicating exactly what variables were used.*
- *I suggest that the results section could include some quantitative results. *

We changed the formulation slightly to “in which contexts which of the variables” should be used. Listing the variables and which of them have which influence here, would exceed the scope and length of an abstract. We have, however, as you suggested, added quantitative information in the results section. Introduction:  

- *"In this paper": I suggest that the authors add further details to this introduction, listing down all if not some of the star classes which are used in this work. *

We have added a couple of sentences detailing the choice of stellar classes and our reasons for selecting them in the introduction.

- *At the end of this section, the objectives of the paper are still not clear despite the general idea being given. I recommend adding a paragraph to list down in 2 to 3 points the exact objectives after the "In this paper" paragraph.*

We have added a specific list of objectives to the end of the introduction *Methods:*  

- *"solar system": capital letters.*
- *PySSED: please include some references to the usage of PySSED in other work.*
  - *Added “An example of PySSED on JWST data can be found in Wesson et al. [24]”, to the manuscript, citing an existing work using PySSED (https://ui.adsabs.harvard.edu/abs/2024RASTI...3...89M/abstract).*
- *"augmented the main dataset": how was this done? What kind of methodology did you use to conduct the augmentation, and why only the main sequence stars? Also, are these main-sequence stars added also classified as other stars at the same time?*
  - We extended the dataset with single label main sequence stars. The reason for only choosing these is due to the abundance of main sequence star data and ease of labelling them. They are sufficiently common such that no one bothers to label them. This cannot be said for the other classes however.
    We added a sentence of clarification to the Extended Dataset section:
    “We only chose to extend this class, due to the abundance of main sequence star data, which is largely left unlabelled due to being sufficiently common.”
- *Figure 1: this plot is not very informative due to the overlapping of the dots, therefore it is difficult to assess the distribution of each type of star. I suggest that the authors plot a 3 x 3 plot, of luminosity vs effective temperature, and each plot for one class only, plotted as a density plot (e.g. three circles indicating the the 68, 97, 99 percentiles based on the concentration of dots per area). With the density plots in place, you would not need to worry about the outliers on the top left as they would not be encapsulated in the plot.*
  - The main point we hoped to convey in Figure 1 was the artificially created outliers, however seeing as the H-R diagram is highly informative for astrophysicists, we have taken your suggestion to heart and added the density plots to the manuscript, see the new Figure 2 .
- *Another info I would like to know from Figure 1 is if the Augmented Dataset for main-sequence stars will look different from the main dataset. Could you visualise this in your updated Figure 1, or explain if there are no differences in the paragraph. This may heavily affect your results section.*
- *"Several deficiencies in the input data": the number and names of input data has not been introduced prior to this point. I suggest that such a discussion on the list of inputs should be placed before this paragraph, or a reference to a section dedicated to the discussion of the inputs.*
  - We have reformulated the sentence to “Several deficiencies in the PySSED output data are apparent.” to make it clear that we were referring to the PySSED outputs. The input features for the classifier as discussed in the PySSED section and in the preprocessing section.
- *"Therefore, finally, ": remove 'finally'.*
- *Train test split: please use a grammatically correct heading.*
- *"442 feature columns": what are these features exactly? Please summarise this using a table. You can shorten the grouped features, e.g. saying that columns 1-200 is the original photometry in wavelength (unit used). Also indicate the percentage of missing values here (which will help the readers understand what is going on later).*
  - Upon careful consideration of your suggestion, we believe a further table could lead to more confusion for the reader, as table 3 already lists the variables used. For readers who like to get the gist of the paper utilizing the table 3, provides better understandability, rather than listing something that is in turn not going to be used.
    In line with providing the most complete data description possible on the default parametrization, we have now clarified that we are using the default output in the text, with reference to the original PySSED paper, which explains the default setup in detail: “Description of the default outputs and bands used are given in \citet{mcdonaldpyssed}.”
    As for the units, although the tree based model we employed is invariant to feature scaling and therefore works irrespective of which unit is used, we added these in table 3.
    To address the final point raised, we added “… , which describes the 74\% total missingness in the main dataset”.
- *Equation 1: please be consistent in your choice of epsilon (ϵ/ε).*
- *"96 features are retained": it is very unclear how this is obtained, please explain in detail how you have reduced 442 features to 96 features. If you need, use figure to visualise the condensation of features to the final number.*
  - We’ve added “After applying the above mentioned preprocessing steps to the default PySSED output data, …” to clarify how the reduction has taken place. Furthermore, we added a short section to the start of the preprocessing paragraph to explain one of the goals: “… , to reduce the data dimensionality”
- *"Figure 2b and Figure 2b": repeated figure here.*
  - We have rewritten the caption and changed the titles accordingly. This should now be much clearer for the reader. Unfortunately, the two plots cannot be collapsed into one, as the x-axis is different, i.e. after preprocessing less features exist to do the missing value analysis on and would make comparison of the missingness harder.
- *Figure 2(a): could you also show the distribution of the augmented data set? Could be in the same plot, with a fainter colour in the background. This is related to the request on augmented data set earlier on. Also, this distribution is not going to train well. Imagine trying to classify massive stars with only 0.24% signal and 99.76% background, how did you expect the machine to differentiate the signals from the noises?*

We added the distribution of the extended dataset to the plots as you suggested. Thanks for pointing this out. We think there might be some confusion between the concept of signal to noise on the one hand, and the ability of a machine learning classifier to learn to separate two (or more) classes. Even if we have a very imbalanced dataset, the majority class cannot be seen as “background noise”. Instead, the majority class is indeed a class. The task of the classifier is to (directly or indirectly) learn the distribution of the features in the input feature space, dependent on the target class, including the majority class. While highly imbalanced datasets can pose a challenge, there is no principal limitation to train on such a dataset. Specifically, the *ratio* between the majority and the minority class is not the main issue at hand. The main issue is the *total* amount of samples of the minority class. What needs to be done when training with imbalanced datasets is to carefully consider the type of training algorithm, and even more so, the correct type of loss functions. Adding even more samples of the majority class would – given the correct choice of training algorithm and loss functions – normally not deteriorate the skill on the minority class, as, once again, this is not an issue of signal to noise ratio, but of learning distributions in the input feature space. This can also clearly be seen in our results from the experiment where we systematically remove samples (figure 6b), and where we found that removing samples from one class – also majority classes - has little to no impact on the classification accuracy on other classes. Additionally, we compared our standard dataset with another dataset (termed augmented dataset in the old manuscript, and extended dataset in the new manuscript) where the prior minority class has been – via inclusion of additional samples - turned into the new majority class. This has not reduced the performance on the other classes. Indeed, the performance was clearly increased (cf. fig 4 and fig. 5). This clearly shows that signal to noise between minority and majority classes is not the issue here, and that increasing the number of majority class samples does not make correctly classifying minority stars harder. Please also note that there is no a-priori way of telling whether a dataset is too imbalanced, or whether the smallest class has – in total numbers – enough samples for successful training. Indeed, one of the main objectives of our study was to find out whether training with the given dataset is possible, and to get indications on how different classes are impacted by their specific number of included samples. Prompted by your response, we have added the following to the Machine-Learning Methods section: “… Herein, models are tasked with either explicitly or implicitly learning some approximation of the sample data's class distribution. A common approach is to have models learn decision boundaries with the aim of separating the support sets of the class distributions, which ideally are aimed at generalizing from the sample to the population. …”  

- *Figure 2(b): it is unclear what this plot is meant to describe. The y-axis label, do you mean it to be "Percentage of data WITHOUT missing values (%)" or "Data Completeness (%)"? If yes, change this, and make it in terms of percentage (0-100%) instead of 0 to 1. Your x-axis, is it cumulative? Are you saying that, e.g. 50 features have 10% missing values, or are you saying that feature #50 has 10% missing values? For either case, the label "Number of Features" does not describe what you are plotting. Please improve the labels. You could also indicate in the plots which is before and after processing, instead of relying merely on the caption to do so.*
  - Thank you for pointing this out. We have now changed he y axis label and the title to clarify that we show a fraction. With the new labels “Number of Features” should now make sense.
- *Table 3: add a column to show the number of input variables in that row, then a row at the bottom indicating the total number of variables.*
- *Change "Other Physics" to just "Physics", keep the consistency throughout the paper.*
- *Table 4: you can condense this table by combining Class Weights 0 to 8 into one single row. Also, please add an extra column to briefly define the hyperparameters and the class weight (or explain them in the paragraph if it takes too much text).*
  Thank you for the suggestion, indeed condensing the table as you have suggested makes a lot of sense, we have taken the appropriate measures and reduced the table size. The hyperparameters and weights are described in the paragraph above the table.
- *Equation 3: while you are at it, you should as well define accuracy here, for completeness sake. You can show the equation when you first mentioned accuracy.*
  - Done

 
*Results:*  

- *You can remove the quotation marks for "Main" and "Augmented", as you have defined them earlier.*
- *"We start by analyzing the skill": replace 'skill' with 'performance' please, do this for all instances.*
- *Figures 4b-f: Could you add a column before precision, indicating the number of objects in each of the classes for this test set that you work on, so that we can assess the results better and dictate if the sample sizes have affected your results.*
  Done. We have added a table with the sample counts per class for the test sets.
- *"The reasons for this remain unclear": it is rather obvious actually, since you only have two variables in the Base model, which are related in a certain way (for blackbodies L ∝ T^4), giving you essentially only one independent parameter to predict 8 different classes. Therefore it is not a good 'model' to begin with. Notice also from Figure 1, how small a range in size (R) your stars are, this further makes it difficult to differentiate 8 classes with 2 seemingly dependent parameters. You really should plot the density plots for Figure 1 I suggested, and look back at this results again and see if you can explain this better.*
  - The referee’s logic here is unclear to us. Indeed, luminosity, temperature and radius are related by the Stefan-Boltzmann law, but the reduction to one independent parameter only works if a tight correlation exists between two of the parameters. While the main sequence represents a partial correlation, we should still expect to see massive stars as the only objects at its end, so even a one-dimensional discriminator should be able to robustly identify them.
  - Massive stars are hot stars, therefore subject to measurement uncertainty in their Wien tails due to lack of UV photometry, which (especially with reddening corrections) cause the unphysical tail of stars noted in the first panel of Figure 1. This measurement uncertainty could be the cause of the lack of ability for the Base (H-R diagram) features to fit massive stars, but unfortunately this is difficult to assess, as it requires both judgement of which stars have been fit in error and a recomputation and retraining of the model.
- *If you really want to find out the effect of the bias package on young stellar objects, I suggest that you plot the RA and DEC of all the classes of stars and code them by colour, and find if you see any patterns. This could be hard evidence to backup your claim here.*
  - The plots have been added to the appendix, see Figure 10.
- *The pool of double-stars observed might be biased, since you require really good resolution to distinguish them from blended objects, and since they are orbiting each other, may give rise to visible traits in the spectra. Therefore, double-stars which are far away or too close to one another may be thought to be just single stars. You should include this fact in your discussion.*
  - The list of double stars at SIMBAD is dominated not by component separation in visual binaries, but by spectroscopic binaries, eclipsing binaries and various types of interacting binaries. It is precisely our hope that these have visible traits in their SED that we can exploit to train the classifier.
    We added this description to the manuscript to clarify it for the reader:
    “This result shows that visible traits in the SED can be exploited by the classifier, as the SIMBAD database is dominated by spectroscopic, eclipsing and various types of interacting binaries, rather than component separation in visual binaries.”
- *"Augmented" dataset: once again, how did you augment your main sequence, where do these extra 80k stars come from? It wasn't clear from the previous explanation. Data augmentation usually means that you generate data from existing data, e.g. rotating images, or adding Gaussian errors to produce new data from existing data. Is this what you did, or did you just add 80k additional main-sequence stars from SIMBAD on top of the sampled main-sequence stars? If this is so, you are not really 'augmenting' your data, it is merely balancing your data. This should be clarified earlier.*
  - We were not referring to data augmentation in the classical ML sense and can understand how this may seem confusing to readers. The terminology was changed throughout the paper to “extended”, as it is non-random sampling from the database.
- *Figures 5b-f: just like Figure 4, add a column of total number of stars before precision.*
- *"Inspired by this result": this should be a new section with a new header.*
- *"Details see text": please correct this grammatically incorrect sentence.*
- *"we only removed the entry from the class undergoing reduction in the dataset": are you saying that e.g. for an object that is both evolved and variable, since you are reducing evolved, you only set its flag for evolved class to zero (to reduce the number of evolved objects) while keeping the flag for variable to 1 (keeping the number of variable objects), thus keeping the entry but modified its properties? I hope this isn't the case, because if it is, this section's results will be invalidated and should be removed entirely, as you are not supposed to modify the entries like that. What you should have in fact, is to reduce the "noise" instead, try reducing the number of other types of stars, such that when you have a higher signal to noise in your sample, to see if your classifier work. I need more clarification on your methodology here.*

We indeed modified the samples as you described it. Given the objective of our experiment – finding out the impact of systematically removing samples of certain start types – this is a viable approach. For the specific case of stars that have two entries, another approach would have been to drop the star from both classes. Here please note that the number of stars with double classification (e.g. evolved and variable) is very low in our dataset (2.67%), therefore, it is not essential which of the two ways is chosen. Dropping the other classes – as you suggested - would be a very different experiment, and not one that would go towards the objectives our paper. As we explained above, signal to noise is not the issue at hand in such a classification task. Please also see our discussion on signal to noise in our reply to your earlier point above. Our methodology in this experiment is further clarified in our response to your next point.

- *I really don't understand the purpose of Figure 6: what were you expecting to happen other than going to zero the more you cut?*

You are right that the performance of the star type that is dropped will of course go to zero the less samples are included. However, the rate at which this happens is not a-priori clear, but this rate is very informative. For example, the ML algorithm might have reached a plateau, in which slightly reducing the number of samples has no impact (as, in our case, happens for lm*). This indicates that including more samples will likely not improve classification performance. On the other hand, for em*, there is an immediate drop in performance. This, consequently, indicates that increasing the number of samples would likely improve classification performance. Additionally, the data shown in panel b of figure 6 shows the impact on the classification performance of the other star types then the one systematically reduced. Here, again, there is no reliable way of telling a-priori whether there will be a significant impact or not. The insights from these experiments are highly valuable for future development of this (or of similar) classifiers. We have now clarified this in the manuscript.

- *"One possible exception are variable stars": well of course it will, since it is the largest proportion among other star types. Removing variable stars completely will significantly increase the signal-to-noise ratio of the other types, therefore increasing the overall average F1 score.*

We agree that this result is not so surprising, given the overlap between variable stars and other stars. While we already discuss this in the manuscript, we added “This is not surprising, as variable stars tend to overlap with other star types.” to make this clearer to the reader that this is a potentially expected result.

- *mau -> may*

   
*Overall comments:*

- After reading the entire paper, I suggest that instead of focusing on 8 classes, the authors should just focus on the results of the augmented sample with 4 classes only: **, ev*, ms* and v*; all other results just don't make much sense in the context of this work due to the exceedingly small signal-to-background ratio. This paper has a novel objective, but was executed poorly. I recommend major revision and rework on the paper before it is considered for indexing.

  Reducing the number of star types to the four that you suggested might seem reasonable at a first glance and would likely increase the apparent classification accuracy on a classifier trained on it. However, it would be a flawed approach, and not meet the main objectives of our paper (to train a first classifier for all stellar classes). We could filter the train and the test set in a way to reduce the types of stars included. However, then the classifier could not be used on data for which the star types are not yet known, as it would per definition be impossible to do the same filtering there. Also, as we explained above, signal to noise or signal to background ratio is not the issue at hand for training a stellar classifier as in our paper. Rather the issue at hand is the class imbalance and small number of samples of the majority classes. The objective was to find out whether this is the limitation for the classifier or not, and we showed that for the minority classes, this is indeed the case.  Note that there could also be other limitations (e.g. with the given features, it might be impossible to separate certain star types beyond a given accuracy, even with unlimited data). Additionally, as mentioned before, the most important thing is not so much the ratio of minority to majority, but the total number of minority samples, and here it is impossible to a-priori determine how high that number has to be to get a good classifier. Therefore, the result that more samples of the minority classes are needed is not trivial. To sum up, the main objectives are (as now after your suggestion stated explicitly in the introduction): *“(1) to provide a first look at the applicability of ML in classification of the entire stellar range, given only sparse photometric measurements based on a random sample of the SIMBAD database, (2) to answer to which extent the sampling bias and associated class imbalance that is inherent in our problem impact the classifier's performance, and (3) what impact increasing the number of samples of minority  classes in training database has on the performance of the classifier.”* These objectives – especially objective (1) - can only be achieved by including all the star classes. We realize that our presentation was not optimal and could have led to a misunderstanding of our objectives and on the details of our methodology as well as the motivation for choosing said methodology. We apologize for the confusion, and hope that in our revised version this is now clearer to the reader.

# Response

**Published:** 2024-08-14

**Authors:** Seán Enis Cody

**Affiliations:** Know-Center GmbH, Graz, Austria

*The manuscript "Machine learning based stellar classification with highly sparse photometry data" by Cody SE, Scher S, McDonald I, Zijlstra A, Alexander E and Cox N, uses Machine Learning (ML) methods for automatically classifying stars. In general, the obtained accuracies are not high enough for the proposed method to be applied directly in subsequent works. However, the authors are aware of this fact and clearly explain the strengths and limitations of their work. Moreover, they obtain and discuss some relevant results, such as the effect of the different sets of variables (bias, base, spectra, physics) on the classification process, which could be very useful in future attempts of automated star classification. Although, as stated by the authors, their work may be considered as a "first step" in studying the feasibility of using ML techniques for star classification, the obtained results and detailed analysis are a significant contribution to the field and I recommend its indexing.* *There are some minor comments that I would like the authors to consider because they could be helpful (clarifying) for any potential reader.*   Dear Reviewer, first of all, we would like to extend our gratitude for taking your time to review our paper and offer feedback. We are also delighted that you recommended our work for indexing and have tried to address your comments as best we could.  

- *The issue of the distance to each source... PySSED determines the distance of each star and then corrects photometry for reddening and estimates temperatures and luminosities. However, estimating distances for large amounts of data is a complex issue, and this may be an important point of uncertainties and/or biases in the results obtained. Distances in PySSED come from different methods, not only parallaxes from Gaia, and even if distances came only from Gaia there may be significant errors or biases coming from the parallax uncertainties. It is a topic that involves PySSED, of course, and it is beyond the scope of this work, but this (potential) drawback should be clearly and explicitly mentioned because it may be one of the factors contributing to a worse performing for the "base variables" set (which includes luminosity).*

**Answer:** The PySSED technical paper is now published, which covers many of these issues. We have referred to it in the text in this context, and expanded relevant comments in the Data section.

- *In order to be clear with potential readers, the fact that the method used throughout this work is XGBoost should be mentioned in the abstract (methods), because it seems that different ML methods are going to be used. Something similar happens with the justification of using only a randomly selected subset (10%) of the complete SIMBAD sample. It is first mentioned just before the conclusions that it is due computational limitations. It would be helpful to know it in the "data" section, especially because the lack of data for a proper training in some subsets is likely one of the reasons for relatively small accuracy values.*

**Answer** **:** Thank you for pointing this out. We have added parts both to the abstract and the data section, clarifying that we are using XGBoost throughout our work and also that the data is only 10 percent due to computational cost.

- *In the abstract-conclusions, the sentence saying that achieved accuracy does not yet allow the classifier to be used by "non-experts" does not seem appropriate. It would be better to state, as in the conclusions, that it cannot yet be reliably used for star classification, or something similar.*

**Answer:** We agree that the formulation was not optimal, and we have changed this part of the abstract conclusions to “not high enough to be reliably used in stellar classification”.

- *Finally, I noticed two typos. When introducing Table 3, it says "... are summarized in in Table 3". And shortly before the conclusions, it says "... skill increase mau suggest that if...".*

**Answer:** Thank you for mentioning these, both typos have been corrected.

# Response

**Published:** 2024-08-19

**Authors:** Seán Enis Cody

**Affiliations:** Know-Center GmbH, Graz, Austria

I think the paper presents a sound and thorough discussion on how machine learning can be used to infer stellar classes. It clearly highlights the importance of having an automated tool capable of classifying large number of sources (stars in this work) in a time-efficient way. The work addresses the technical challenge of such a goal and describes how the performance of the classification can be improved in the future. The work shows promising results. I believe that the paper is clear and well written, and the scientific analysis is interesting and carried out in a rigorous way. I therefore recommend the paper for indexing. I have a however few suggestions that I believe could improve the paper before it is indexed. I understand that addressing all these suggestions may not be feasible, but I would appreciate it if the authors could consider them and provide their thoughts. *Dear reviewer, thank you for taking the time to review our work and for providing us with valuable feedback! We’re happy to hear that the paper was well received. We have tried our best to address and incorporate your feedback into the revised paper. Please find detailed responses to all your comments below.* **Main comments:**

- While I think that the authors did a great job in addressing possible technical issues and sources of bias, I believe that some astrophysical aspects may have been overlooked. Specifically, there is currently a wealth of information for millions of stars in the Milky Way, and many surveys provide accurate spectro-photometric information (see e.g. SDSS-17, APOGEE, LAMOST DR9, GALAH DR3, Gaia DR3).
  These are only some of the datasets available today, and more complete and numerous surveys are planned to be released and/or carried out in the forthcoming years.
  Therefore, I believe that more information could be exploited to infer stellar types. Additionally, I wonder whether using physical variables coming from different datasets (e.g. Teff, logg from actual spectroscopic surveys, instead of from SED fitting) might improve the performance of the classification, while avoiding the SED fitting step. Indeed, SED fitting is highly sensitive on the de-reddening procedure and accurate estimates of absorption are often required to produce reliable results.
  Additionally, could the ML algorithm be trained using the same classification, but exploiting Gaia XP spectra (or stellar properties derived from XP spectra, like for example in Andrae *et al.*, 2023 ^1^ )? This could drastically increase the sample of stars to which the algorithm could be applied, while possibly increasing the accuracy of the classification (for some or all classes).

**Answer:** *The aim of our approach was to discern whether we could identify stellar types from photometric data alone. We agree that a large number of spectra exist, especially from Gaia, spectral and spectrophotometric means of establishing stellar types are indeed better. However, software to establish stellar types from such data already exist, and (despite the millions of spectra available) the vast majority of stars still lack spectra (e.g. only 32% of Gaia DR3 stars have spectra).* *A major driver behind creating this software is so that we have a classifier that can work in the absence of spectroscopy, both to provide an independent classification to spectral-based classifiers, and to have a procedure we can use among stars where spectra cannot be taken, including deeper observations of the Milky Way and external galaxies that are being taken by (e.g.) Euclid, Roman, Rubin, HST and JWST. We have highlighted this in the introduction and return to this point further down in our response.* *Dereddening is an issue that affects the accuracy of our fitting. In the original PySSED paper (the cited McDonald et al. 2024), the dereddening procedure was demonstrated to be robust in the majority of cases though (as Figure 1 shows) there is an asymptotically increasing sensitivity as stars become hotter. Since the dereddening procedure uses Gaia BP/RP spectrophotometric data Gaia spectrophotometry can no longer be treated as an independent result for comparison: we have highlighted this in the paper. Regardless, one item we aim to measure is the circumstellar reddening around many stars, which gives a clue to their categorisation.* *In essence, we consider the main limitation that limits the accuracy of our approach is that the types of star that we have tried to model (SIMBAD’s top level classifications) cannot all accurately be identified from SED fitting.*   I am not sure the “Preprocessing” section is entirely clear. Specifically, the authors mention they retained 96 features, of which 85 belongs to the Spectra variables. Does this mean that for each star there are up-to 85 photometric measurements? If that’s the case, I think it could be informative to understand how missing values among photometric data affect the predictive skill of classes. Specifically, having less photometric information would affect the outcome both because of less information is available to the algorithm, and because the SED fitting might underperform. Therefore, could there be a minimum number of photometric bands (or a minimum number of photometric bands per wavelength region) to ensure the best performance without compromising the sample size? Additionally, could the predictive skill of different classes be dependent on the typical number of available features of that class?

**Answer:** *Indeed, in our scenario, a star can have measurements within 85 different bands, which leads to a high level of sparseness in the data. As part of our early preprocessing steps we also included a feature selection based on low variance bands, under the assumption of low variance = low information. We thereby hoped to improve generalization, however the results did not improve, as the ensemble tree structure is already performing an internal feature selection through the tree-splitting criterion. In other words, it made sense for us to keep all features in, as XGBoost would use a feature only in making decisions if the internal “gain” is great enough (if truly all features with low variance were worse than the other more densely packed features, their impact on the classification would be negligible).* *The quality of the SED fitting in the majority of cases does not strongly depend on the number of photometric datapoints, but rather on their distribution in wavelength. A surprisingly good fit is normally achieved even if only a handful of the 85 possible bands have been observed (see the cited McDonald et al. 2024). In most cases, the majority of the impact from sparse data is therefore on the data available to the classifier for predicting classes.* *As for the predictive skill of classes being tied to sparseness of its bands, we utilized SHAP importance measures to investigate this further.*

*At first glance our findings seemed to align with the natural assumption, that features with more entries contained more information. For example, for the Physics Feature set, we found that 18 out of the 20 features that contributed most to the classification were among the 20 most densely populated features. Interestingly however, the WISE/WISE.W4 band which was the most impactful feature overall was not among these. In fact, upon observing the samples most impacted by this band via local explainability, we found that out of the top 50% most impacted main sequence star samples, 95% were missing values. Similarly, the top 5% of samples impacted most by this band, had over 92% missingness. This indicates that the predictive performance of different classes is not entirely dependent on the typical number of available features of that class, but rather that missing value patterns can be learned to act as discriminative features using XGBoost’s default pathway choices.* *We have added a brief section at the end of the results section, right before the conclusions, in response to this question.*  

- On a similar note, if the missing photometric measurements fall into the Gaia XP spectra wavelength region (350-1000 nm), could Data imputation be a feasible alternative? Indeed, one could substitute missing photometric values with synthethic photometry generated by means of calibrated Gaia XP spectra (see e.g. Gaia Collaboration, Montegriffo *et al.* 2021 ^2^ ). Can the authors comment upon this?

**Answer:** *While this could certainly be done in principle, we are (as mentioned above) primarily interested in identifying the stellar classification that can be performed with photometry alone, as it has wider applications beyond the subset of objects for which spectra are available.*

  

- Finally, I believe that not all photometric magnitudes carry the same weight. Are there specific photometric features that affect the predictive skills more than others? Is there a dependence on specific class (e.g. Is the infrared more relevant for the predictive skill of some classes?)

**Answer:** *Yes, some wavelength bands are more important than others. We also investigated this using SHAP importance (see updated Fig. 8) and found that specific wavelengths were more indicative for some classes. In many cases this increase in predictive skill correlated with the abundance of sample entries at that specific wavelength. We did however also find highly sparse wavelengths, which had missing values, that were used to make reasonable predictions. This is largely to do with XGBoost’s default pathways for missing values providing the capabilities to use missing values as discriminative features values. The Result for the Full feature set is shown in R1 for the top 20 most important features in the sample extended dataset, with class specific importances highlighted by varying colour. We have added the plot and a brief discussion on this to our results section of the paper, right before the conclusions.*

  

- I did not understand why the authors randomly selected 10% of the whole SIMBAD sample of classified stars. Does it only refer to the training, or the remaining 90% has not been used at all? Does including all the stars improve the efficiency of the ML classification or it has negligible effect? Is it a choice motivated by computational costs? If so, I believe it could be better expressed in the Data section.

**Answer:** *Only 10% of SIMBAD’s classified stars were used as inputs to the classifier due to the requirement that these stars were first pre-processed by PySSED. Processing a list of stars this way takes a long time due to the database setup at CDS and the multiple queries per star that need to occur as a result. Hence, the remaining 90% of stars haven’t been used at all.* *In the discussion surrounding Figure 6, where we dropped samples per class, we noted that the problem is likely rooted in the underrepresentation of minority classes and not in the class imbalance itself. Therefore, as you have surmised, any increase in data leading to an increase in minority samples is likely to improve the classifier’s performance.* *We realize that this was not made clear enough in the manuscript and have edited the data section accordingly. We added “, the choice of which was largely dictated by computational time, originating from evaluating multiple queries per star at the CDS SIMBAD database during the PySSED preprocessing steps” after specifying the 10 percent sample size.*  

- I believe it would be informative to better show the H-R diagrams of the input sources. Indeed, it is quite difficult to visualize the different classes from the right panel of current Figure 1. Could the authors show different classes in different panels. Specifically, the H-R diagram is a highly informative tool which allows to disentangle different classes of stars. Naively, I expect that the algorithm will perform better for stars in distinct HR diagrams location, as also mentioned by the authors in their discussion. On a similar note, I believe it could be useful to show the HR diagrams of correctly/incorrectly classified stars. I would ask to see these plots in the referee report, but I leave the choice of including them in the main manuscript to the authors.

**Answer:** *Thanks for bringing this up, indeed it is hard to distinguish the classes in Figure 1. The main message we hoped to convey here was the artificial outliers generated as part of the SED fitting procedure, which in turn ties into our further motivation for the choice of XGBoost or rather the missing outlier removal preprocessing step (as the model is robust to these outliers). Therefore, we had chosen not to add these plots to the paper, as the message we hope to discuss would be harder to identify. We take note however that, as you say, the H-R diagram is highly informative to astrophysicists and have therefore included the requested plots as the updated figure 2.* *Regarding the HR diagrams of correctly/incorrectly classified stars, we expanded this to false positives, false negatives and true positives and added it to the appendix, see updated Figure 9.*

  

- Would it be possible to test the predictions of the algorithm on unclassified data? Specifically, stars clusters, such as globular clusters and open clusters, could represent a valuable source of information. Indeed, while it would not be possible to test all classes, it would be quite straightforward to test the classification of low and high-mass stars, main-sequence stars, and evolved stars. There are currently multiple catalogues of cluster members based on Gaia DR3 (Cantat-Gaudin *et al.*, 2020 ^3^ ; Tarricq *et al.* 2022 ^4^ ; Hunt *et al., *2024 ^5^ ) that could be used to this goal. Can the authors comment upon this?

**Answer:** *This is a very sensible suggestion. There is substantial additional science that can come from analysis of both globular and open clusters with SED fitting (e.g.               2011ApJS..193...23M, 2009ApJ...705..746B, 2009MNRAS.394..831M). For this reason, we would prefer to perform this kind of analysis as part of a separate study focussing on SED fitting of globular clusters in general, and we have applied for funding to undertake this.*

  

- Would decreasing the number of classes improve the efficiency of the classification? Would removing the most under sampled classes affect the training and outcome? Specifically, as the authors mentioned that sparsity and heterogeneity if the input sample represent important challenges, I am wondering if a more homogeneous distribution among classes would help improve the efficiency of the classification. Indeed, as this is a first attempt at using ML to classify stars, I believe it would be more important to increase the efficiency of the classification of a subset of classes, rather than including all classes. If having a more homogeneous training set does not affect the outcome, that the authors can disregard this last comment.

**Answer:** *The choice and number of classes discussed in this paper is certainly sub-optimal, due to constrains on time and funding for this work. However, we felt it important to publish those components of research we could optimise, in order that the community could take this research forward in future.* *The broad SIMBAD classes that we examine each contain a heterogeny of different types. For example, many classes of variable star (e.g. novae sources, T Tau stars or Mira variables) could be easily identifiable due to infrared excess caused by circumstellar dust, whereas other classes (e.g. eclipsing binary stars or spotted stars) may be very difficult to identify from SED fitting.* *Ideally, we would like to progress through the entire SIMBAD hierarchical tree of classes, identifying those where our classifier can obtain results with a high degree of confidence (F1 score), and creating a (potentially much larger) set of classes to choose between, each of which could better inform the classes of stars further up the hierarchical tree. However, that level of decision and subsequent optimisation is a corpus of work in its own right. *

- Would training an algorithm to classify only one class at a time improve the accuracy of the classification? Or it would not affect the predictive skills? Do the authors have any opinion on that?

**Answer:** *In our experiments this was not the case, we also briefly studied a one vs rest (OVR) approach, where the result was based on a set of binary classifiers, however the results were quite similar. We did this largely based on results from the literature (https://www.aanda.org/articles/aa/pdf/2022/10/aa43135-22.pdf)* *, pertaining to advantages of OVR in stellar classification. Since the results were similar enough though, we avoided this approach in subsequent steps, as the final model obtained with an OVR approach would also have been more complex to explain. We therefore retained the multiclass model approach. *

- This comment is partially connected to comment #1. Would excluding the base variables (i.e. physics – base) strongly affect the results? If so, in what way? As the authors show that the base model generally has a lower skill, I wonder if that could be a consequence of a non-reliable SED fit. If that is the case, is it possible that removing the base model would improve the predictive skill? Additionally, as the spectroscopic temperature is already included in the “other physics” model, one would only use the information about the luminosity of the star. Could the authors comment upon this? Furthermore, would removing the SED fit step reduce the computational cost? If so, would this allow to increase the training sample size?

**Answer:** *There are two categories of information encoded by the SED fit. The first are the properties of the SED fit itself, which forms the Base features. The second are the discrepancies between the SED fit and the stellar atmosphere model used to create the fit.* *Removing the Base variables is possible, but essentially this removes information about where on the H-R diagram a star sits. For example, this removes much of the ability to distinguish between main-sequence and evolved stars, since the effects of surface gravity on SEDs are relatively minor. These Base variables are correspondingly some of the most critical in fitting stars (as identified by their high SHAP importance). Again, only a fraction of stars have spectroscopic temperatures, so we do not wish to rely on it.* *Removing the SED fit entirely is not possible due to the second category of information. In essence, we fit the difference between the stellar model and the star itself. Without the SED fitting, we remove not only the astrophysical parameter generation, but also the extinction correction, the removal of erroneous photometry, and the model normalisation. Since the computational bottleneck is simply in obtaining the basic photometry for each star from CDS, removing the SED fit come at a significant cost without any significant benefit to computational speed.* **or comments:**

- At page 7, in the first lines, Figure 2b is repeated twice. Could the second instance be Figure 2c?

**Answer:** *The differences are minor, as the shape remains the same, however the first is the unpreprocessed data and the second post spectra transformation. The message conveyed is more or less identical, yet for completeness' sake, the non-preprocessed raw data is also shown. To make this clearer to the reader we have now adjusted the titles of the plots. Furthermore, we have added the extended dataset to these plots.*

  

- I think it could be useful to mention which photometric data is being used in the SED fitting, or at least give few examples of the SED fitting process for stars with large/medium/low available photometric information, and maybe how the retrieved parameters (Teff and log) compare with spectroscopic measurements, if available.

**Answer:** *Examples of this are given in the PySSED technical paper, which is now published. We have added text to the PySSED section to reference this paper and discuss these issues in this context.*
